# Virus infection of *Haptolina ericina* and *Phaeocystis pouchetii* implicates evolutionary conservation of programmed cell death induction in marine haptophyte–virus interactions

**DOI:** 10.1093/plankt/fbu029

**Published:** 2014-05-05

**Authors:** Jessica L. Ray, Liti Haramaty, Runar Thyrhaug, Helen F. Fredricks, Benjamin A. S. Van Mooy, Aud Larsen, Kay D. Bidle, Ruth-Anne Sandaa

**Affiliations:** 1 Uni Research As, Thormøhlensgt 49B, N-5006 Bergen, Norway; 2 Department of Biology, University of Bergen, Thormøhlensgt 53A, N-5006 Bergen, Norway; 3 Institute of Marine and Coastal Sciences, Rutgers University, 71 Dudley Road, New Brunswick, NJ 08901, USA; 4 Marine Chemistry and Geochemistry, Woods Hole Oceanographic Institution, Woods Hole, MA 02543, USA

**Keywords:** caspase, DNA fragmentation, IETD, Phycodnaviridae, z-VAD-fmk, haptophyte

## Abstract

The mechanisms by which phytoplankton cope with stressors in the marine environment are neither fully characterized nor understood. As viruses are the most abundant entities in the global ocean and represent a strong top-down regulator of phytoplankton abundance and diversity, we sought to characterize the cellular response of two marine haptophytes to virus infection in order to gain more knowledge about the nature and diversity of microalgal responses to this chronic biotic stressor. We infected laboratory cultures of the haptophytes *Haptolina ericina* and *Phaeocystis pouchetii* with CeV-01B or PpV-01B dsDNA viruses, respectively, and assessed the extent to which host cellular responses resemble programmed cell death (PCD) through the activation of diagnostic molecular and biochemical markers. Pronounced DNA fragmentation and activation of cysteine aspartate-specific proteases (caspases) were only detected in virus-infected cultures of these phytoplankton. Inhibition of host caspase activity by addition of the pan-caspase inhibitor z-VAD-fmk did not impair virus production in either host–virus system, differentiating it from the *Emiliania huxleyi*-Coccolithovirus model of haptophyte–virus interactions. Nonetheless, our findings point to a general conservation of PCD-like activation during virus infection in ecologically diverse haptophytes, with the subtle heterogeneity of cell death biochemical responses possibly exerting differential regulation on phytoplankton abundance and diversity.

## INTRODUCTION

Marine phytoplankton account for a large proportion (∼50%) of global primary production and have a strong influence on global nutrient cycling (Field *et al*., 1998). Knowledge of mechanisms regulating phytoplankton mortality is therefore essential in order to gain a better understanding of marine ecosystem function and the fate of fixed organic carbon. The classically implicated causes of phytoplankton mortality include predation by herbivores (Landry and Hassett, 1982) or infection and lysis by virus (Bratbak *et al*., 1993; Fuhrman, 1999). As the most abundant biological entities in the ocean, viruses are a major cause of phytoplankton mortality (Suttle, 2005, 2007) and can lead to enhanced upper ocean respiration (Fuhrman, 1999). More recently, programmed cell death (PCD)-like pathways have been shown to mechanistically execute phytoplankton mortality in diverse phytoplankton species (Berges and Falkowski, 1998; Vardi *et al*., 1999; Lawrence *et al*., 2001; Berman-Frank *et al*., 2004; Bidle *et al*., 2007; Ferroni *et al*., 2007; Allen *et al*., 2008; Bidle and Bender, 2008; Thamatrakoln *et al*., 2011; Franklin *et al*., 2012; Franklin, 2013), thereby implicating PCD as an important contributor to the turnover of primary production and nutrient recycling in the world's oceans. A clear mechanistic link has now been well established between host PCD and viral infection for the coccolithophore *Emiliania huxleyi*–Coccolithovirus system, for which seasonal algal blooms are consistently terminated by virus infection (Bratbak *et al*., 1993; Wilson *et al*., 2002). EhV infection not only triggers the host PCD biochemical machinery, but also recruits it for successful virus production (Evans *et al*., 2006; Bidle *et al*., 2007; Vardi *et al*., 2009; Bidle and Vardi, 2011). PCD-like markers such as chromatin condensation, reactive oxygen species (ROS) production and cysteine aspartate protease (caspase) activation have also been observed during virus infection of other phytoplankton (Lawrence *et al*., 2001; Evans *et al*., 2006; Bidle *et al*., 2007). These markers are diagnostically associated with PCD in metazoans and other unicellular eukaryotic organisms (Ameisen, 2002; Bidle and Falkowski, 2004; Franklin *et al*., 2006; Bruchhaus *et al*., 2007). In light of the widespread, and nearly universal distribution of PCD-related genes, including numerous caspase-family proteins (i.e. metacaspases), in the genomes of diverse lineages of photoautotrophs (Bidle and Falkowski, 2004), these findings suggest that PCD may play a pronounced role in haptophyte–virus interactions.

We performed experiments to determine the extent to which the *E. huxleyi*–EhV paradigm is relevant for other marine haptophytes for which similarly large, double-stranded DNA containing viruses (Phycodnaviridae) have been isolated and characterized. We investigated cultures of the single-celled marine haptophytes *Haptolina ericina*, a cosmopolitan non-blooming phytoplankter (Sandaa *et al*., 2001), and *Phaeocystis pouchetii*, a colony- and bloom-forming phytoplankter found in high-latitude oceans (Jacobsen, 2002; Schoemann *et al*., 2005), using diagnostic molecular and biochemical markers for PCD activation. Lytic viruses infecting *H. ericina* (CeV-01B) (Sandaa *et al*., 2001) and *P. pouchetii* (PpV-01B) (Jacobsen *et al*., 1996) have been isolated from Norwegian coastal waters and are well studied in laboratory cultures. These haptophyte–virus systems provided a unique opportunity to assess the expression of PCD-like traits during virus infection in these haptophytes, and thereby contribute to the greater body of knowledge concerning haptophyte–virus interactions in the marine environment.

## METHOD

### Phytoplankton cultures

*Haptolina ericina* (Parke & Manton) and *P. pouchetii* (Jacobsen *et al*., 1996) were obtained from the algal culture collection maintained in the Department of Biology at the University of Bergen. Cultures were grown in IMR/2 medium (Eppley *et al*., 1967) at 15°C and 8°C, respectively, at 180 µmol photons m^−2^ s^−1^.

### Viral propagation

The double-stranded DNA viruses CeV-01B (Sandaa *et al*., 2001) and PpV-AL02 (A. Larsen, unpublished results) were maintained at 4°C in the dark with regular propagation in *H. ericina* and *P. pouchetii* host cultures, respectively. Fresh virus lysate was prepared by inoculating an exponential-phase host culture with 1% (v/v) virus lysate to give a multiplicity of infection (MOI) of ∼10 viruses per host cell. Cultures were incubated for 4–5 days until lysis was apparent. Viruses were harvested from culture lysates by two rounds of centrifugation at 5445 × *g* at 4°C for 20 min, followed by passage through a sterile 0.45-µM pore-size cellulose acetate syringe filter to remove cellular debris and bacteria. Half of the volume of fresh virus lysate was boiled for 10 min to inactivate virus particles. This heat-killed virus lysate was used as a negative control inoculum for virus infection. All virus lysates were stored at 4°C for no longer than 1–2 days prior to use in infection experiments.

### Virus infection experiments

**Fig. 1.**
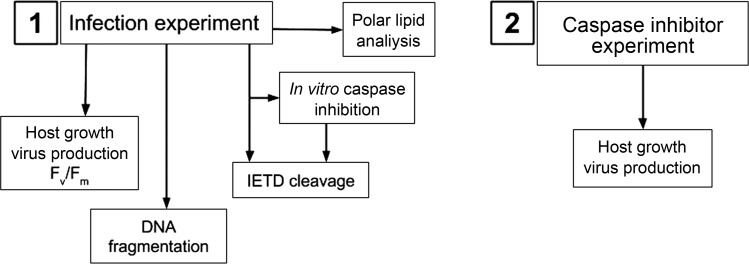
Flow diagram illustrating the experimental design of this study.

Experiments were started when the density of exponentially growing phytoplankton cultures reached 1–2 × 10^5^ cells mL^−1^ after at least 1 week of daily 1:1 dilutions with fresh IMR/2 medium. These pre-experimental dilutions resulted in consistent observations of growth rates of approximately one division per day (data not shown), demonstrating that cultures were actively growing prior to infection. We infected healthy cultures only at the start of the day period in an attempt to emulate culture synchronicity at the time of virus exposure. Cultures were inoculated with either fresh active virus lysate (MOI > 5), heat-killed virus lysate or sterile IMR/2 medium, with each amendment mixed into the culture by gently swirling before dividing equally into three replicate culture flasks. *H. ericina* cultures were incubated at 15°C with ∼180 µmol photons m^−2^ s^−1^ on a 14:10 light: dark cycle, while *P. pouchetii* was incubated with similar continuous irradiance (180 µmol photons m^−2^ s^−1^) at 8°C. Virus, phytoplankton and bacterial cell counts were performed on a FACSCalibur flow cytometer (Beckton Dickinson) according to the methods of Thyrhaug *et al*. (Thyrhaug *et al*., 2003). The maximum quantum efficiency of photosystem II (Fv/Fm) was determined from variable fluorometric measurements of culture samples as described previously (Bidle *et al*., 2007), and was used as an indicator of culture health. A flow diagram of the experimental design is shown in Fig. 1, part 1.

### DNA fragmentation

Electrophoretic examination of genomic DNA extracted from *H. ericina* and *P. pouchetii* cultures was performed to investigate whether apoptotic DNA laddering (Nagata, 2000) occurs during viral infection of these phytoplankton. Culture samples (200–250 mL) were taken at various time points after infection (0, 1, 2 and 3 days post-virus addition). Cells were harvested by centrifugation at 1600–6400 × *g* at 4°C in a Beckman JA-10 rotor. Genomic DNA from cell pellets was immediately extracted using an Apoptotic DNA Ladder kit (Roche Applied Science, Indianapolis, IN, USA) according to kit instructions. After elution in 70°C elution buffer, RNA was removed from samples by the addition of 20 U RNAse A (Promega, Madison, WI, USA) and incubation at 37°C for 30 min. DNA was then purified using the Clean and Concentrator kit (Zymo Research, Irvine, CA, USA) and eluted in a final volume of 20 µL of 70°C distilled water. Four microliters of 6× DNA loading buffer (Promega) was added to each sample, and samples were stored at 4°C in the dark until analysis by agarose gel electrophoresis at 4°C in 1% (w/v) agarose at 100 V for 2 h in 40 mM Tris–Cl, 20 mM acetic acid, 1 mM EDTA, pH 8.0. Gels were stained in 1X SYBR Gold (Invitrogen, Carlsbad, CA) at room temperature in the dark for 1–2 h prior to visualization using a GelDocXR (Bio-Rad, Hercules, CA, USA).

### IETDase catalytic activity

Activation of host cysteine aspartate-specific proteases (caspases) is another hallmark of PCD (Vardi *et al*., 1999; Ameisen, 2002). We examined control and virus-infected cell extracts of both *H. ericina* and *P. pouchetii* for changes in caspase-like catalytic activity during virus infection by measuring cleavage of the flurogenic, canonical caspase-8 substrate isoleucyl-glutamyl-threonyl-aspartic acid-7-amino-4-methylcoumarin (IETD-AMC; 50 µM) (Supplementary data). The kinetics of fluorescence were measured using a Spectra Max Gemini XS plate reader (excitation 400 nm, emission 505 nm) and normalized to total protein [relative fluorescence units (RFU) mg protein^−1^ hr^−1^], as previously described (Bidle *et al*., 2007).

The same cell extracts were used to verify *in vitro* inhibition of caspase activity with the pan-caspase inhibitor z-Val-Ala-Asp-fluoromethyl-ketone (z-VAD-fmk; 20 µM final concentration) (Calbiochem, Darmstadt, Germany). Fresh z-VAD-fmk inhibitor was dissolved immediately before use in molecular biology grade dimethyl sulphoxide (DMSO) to a concentration of 20 mM. Pooled control or virus-infected cell extract samples were pre-treated with 20 µM z-VAD-fmk (Bidle *et al*., 2007) for 1 h prior to IETD-AMC cleavage measurements (as described above). IETDase cleavage activities (RFU mg protein^−1^ hr^−1^) for z-VAD-fmk-treated extracts were normalized to the activity for untreated cell extracts and are expressed as a per cent reduction in IETDase cleavage activity. Total caspase inhibition (100%) with z-VAD-fmk was confirmed on recombinant human caspase-8 (data not shown).

### Polar lipid analysis

Production of viral glycosphingolipids (vGSLs) during infection by the EhV virus has been shown to play a critical role in the regulation of virus-mediated cell lysis in *E. huxlyei* (Vardi *et al*., 2009, 2012). Ten-millilitre aliquots from each of three replicate cultures per treatment (control and virus-infected) were pooled and passed through pre-baked GF/F filters. Filters were aseptically transferred to cryostorage tubes and frozen in liquid nitrogen, thereafter stored at −80°C until analysis as described previously (Vardi *et al*., 2009, 2012).

### Effect of *in vivo* caspase inhibition on host growth and viral infection dynamics

Exponentially-growing cultures of *H. ericina* or *P. pouchetii* were pre-treated with 20 µM z-VAD-fmk prior to infection by their respective viruses, in order to determine whether z-VAD-fmk impairs virus production (Bidle *et al*., 2007) (Fig. 1, part 2). On the day before the experiment started, cultures were diluted 2-fold with fresh IMR/2 medium, and 20 mL aliquots of diluted culture were distributed into each of twelve, sterile, 25-mL glass flasks by very gentle pipetting. Pre-treatments consisted of: sterile IMR/2 medium, 0.5% (v/v) DMSO or 20 μM z-VAD-fmk. Cultures were swirled gently to mix then incubated for 2 h in normal culture incubation conditions. After 2 h, 100 µL of boiled virus lysate was added to one set of pre-treated cultures, and to the other set was added 100 µL of infectious virus lysate. The experiment was set-up with a 3 × 2 factorial design with duplicate cultures for each treatment. Sampling took place immediately after control/virus addition on the start day of the experiment (Day 0) and for three consecutive days (Days 1–3). Samples were taken for flow cytometric determination of algal cell counts and virus and bacteria counts. Count data were fitted to a generalized linear mixed model using penalized quasi-likelihood (Bolker *et al*., 2009) in the MASS package (Venables and Ripley, 2002) in R (Hornik, 2013). Cell or virus counts were modelled as a function of treatment and time (categorical independent variables), and as a function of the interaction between treatment and time, and random effects due to biological and technical replication were also tested.

## RESULTS

### Host growth, virus production and culture dynamics

**Fig. 2.**
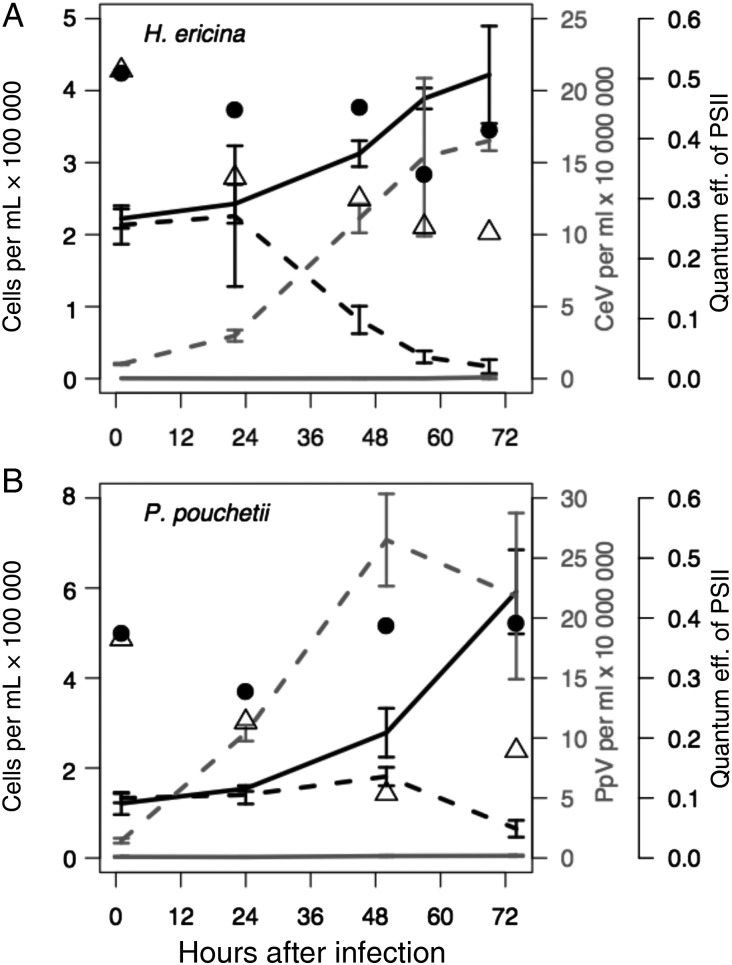
Phytoplankton growth, virus production and quantum efficiency of photosystem II (Fv/Fm) in control and virus-infected cultures of (**A**) *Haptolina ericina* and (**B**) *Phaeocystis pouchetii*. The viruses CeV-01B or PpV-01 were used to infect *H. ericina* and *P. pouchetii* cultures, respectively. Solid black lines, host growth in control cultures; dashed black lines, host growth in virus-infected cultures; solid grey lines, virus production in control cultures; dashed grey lines, virus production in virus-infected cultures. Black circles, Fv/Fm in control cultures; open triangles, Fv/Fm in virus-infected cultures.

Host cell growth in *H. ericina* control cultures increased daily, reaching a final concentration of 4 × 10^5^ cells mL^−1^ on the last day of the experiment (Fig. 2A, solid black line). Growth in CeV-infected cultures continued until 24 h post-virus addition, after which time cell numbers decreased daily until the end of the experiment, when the mean cell concentration was 2 × 10^4^ cells mL^−1^ (Fig. 2A, black dashed line). Cell density of *P. pouchetii* control cultures, increased daily during the experiment (Fig. 2B, solid black line), reaching ∼6 × 10^5^ cells mL^−1^. Host cell abundance in PpV-infected cultures remained stable at 1.5 × 10^5^ cells mL^−1^ until 48 h post-virus addition, after which time it decreased to 1 × 10^4^ cells mL^−1^ by 72 h post-virus addition (Fig. 2B, dashed black line). These results are consistent with previously published studies of *H. ericina*-CeV and *P. pouchetii*-PpV dynamics (Jacobsen *et al*., 1996; Sandaa *et al*., 2001).

Enumeration of viruses by flow cytometry demonstrated positive net CeV and PpV production as soon as 24 h post-virus addition in both experiments (Fig. 1). Virus production in CeV-infected cultures of *H. ericina* (Fig. 2A, dashed grey line) peaked at 1.6 × 10^8^ virus particles at 70 h post-virus addition, while PpV production in virus-infected *P. pouchetii* cultures (Fig. 2B, dashed grey line) peaked at 50 h post-virus addition at 2.6 × 10^7^ PpV mL^−1^. Burst sizes were calculated to be 890 viruses per *H. ericina* host cell and 289 PpV viruses per *P. pouchetii* host cell (Sandaa *et al*., 2001).

Fluorescence measurements demonstrated lower mean Fv/Fm values for virus-infected cultures (open triangles) relative to control cultures (black circles) at all time points after *T*_0_ (Fig. 2), indicative of decreasing photosynthetic health due to virus infection. Fv/Fm values decreased from 0.5 to 0.3 only in CeV-01B-infected cultures of *H. ericina* (Fig. 2A, open triangles), a decrease which was more or less synchronous with CeV-01B production dynamics (Fig. 2A, dashed grey line). Photosynthetic efficiency (Fv/Fm) also decreased slightly in control *H. ericina* cultures, although not to the same extent as the virus-infected cultures (Fig. 2A, black circles). For *P. pouchetii*, Fv/Fm values in control cultures remained stable at 0.4 over the course of the infection experiment, but decreased from 0.4 to 0.2 in PpV-01B-infected culture (Fig. 2B, black circles), indicative of culture crash due to viral lysis (Fig. 2B, dashed black line).

### DNA fragmentation

**Fig. 3.**
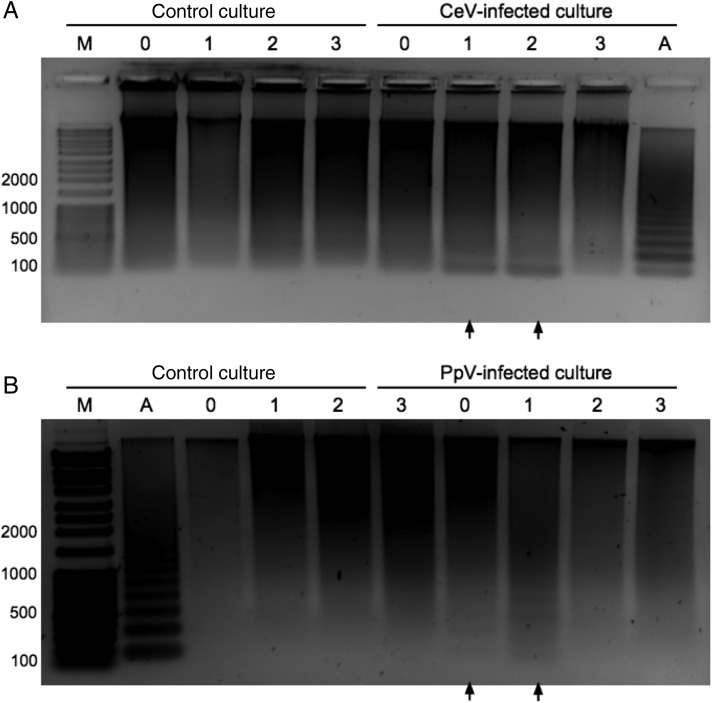
Induction of DNA fragmentation during CeV infection of *Haptolina ericina* (**A**) and PpV infection of *Phaeocystis pouchetii* (**B**) cultures. Lane identifiers: M, dsDNA molecular weight marker with sizes shown in basepairs; A, positive control mammalian U937 apoptotic cells; 0–3, time in days after treatment with heat-killed (control culture) or infectious (CeV- and PpV-infected) virus lysates. Black arrows underneath wells indicate positive identification of DNA laddering.

Notable DNA fragmentation occurred in both *H. ericina* (Fig. 3A) and *P. pouchetii* (Fig. 3B) cultures inoculated with infectious virus (CeV-01B or PpV-01B). Chromatin cleavage commenced in the early stages (Days 0–1) of virus infection in both phytoplankton, and continued to accumulate for at least 2 days post-virus addition. For *H. ericina*, we observed a clear increase in low-molecular-weight (∼200 bp) DNA fragments at 1-day post-virus addition. Characteristic DNA fragmentation in virus-infected cultures was not apparent once cultures had completely lysed (Fig. 3, 3 days post-virus addition in infected cultures). DNA fragmentation in virus-infected cultures of *H. ericina* appeared as the accumulation of a low-molecular-weight DNA band on Days 1 and 2 post-virus addition (Fig. 3A, black arrows). We also observed detectable, weak DNA “laddering” on Days 2 and 3 in the control *H. ericina* culture (Fig. 3A) that was distinct from the DNA fragmentation seen for the virus-infected culture. For *P. poucheti*i cultures infected with PpV-01B virus, DNA fragmentation appeared as a distinct laddering phenotype (Fig. 3B, black arrows). Genomic DNA fragmentation in PpV-infected cultures of *P. pouchetii* was already apparent in samples taken immediately after PpV addition at the start of the experiment (0 h, Fig. 3B). With time allowances for centrifugation and resuspension in lysis buffer, this would indicate that DNA fragmentation in *P. pouchetii* was induced within 30 min after virus addition. The same results were observed for at least two independent experiments for both *H. ericina* and *P. pouchetii*. We observed DNA laddering patterns for *H. ericina* and *P. pouchetii* cultures upon treatment with camptothecin (Supplementary data, Fig. S1), a DNA topoisomerase I inhibitor known to induce replication arrest and PCD (Sen *et al*., 2004), thereby confirming the inducible PCD-like DNA fragmentation phenotype associated with virus infection. Similar DNA fragmentation was not observed after 24 h for untreated control cultures (data not shown).

### IETDase catalytic activity in cell extracts

**Fig. 4.**
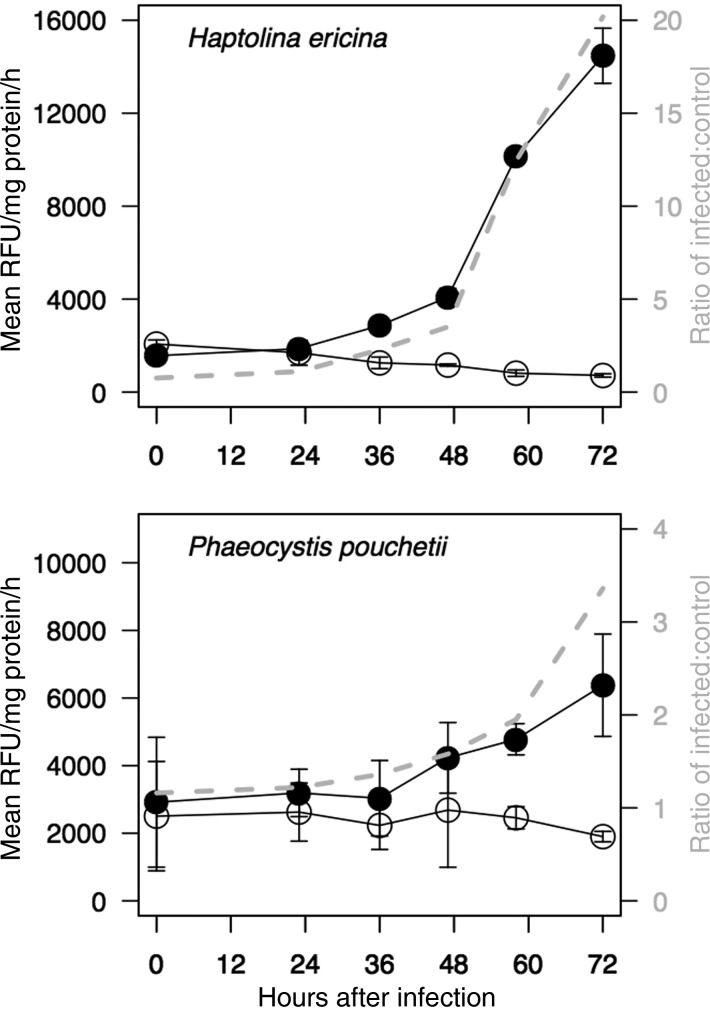
Cleavage of the fluorogenic caspase-8 substrate isoleucyl-glutamyl-threonyl-aspartic acid-7-amino-4-methylcoumarin (IETD-AMC) in soluble cell extracts of control (open circles) and virus-infected (black circles) cultures of *H. ericina* (top) and *P. pouchetii* (bottom). Cultures were infected with CeV-01B or PpV-01B viruses, respectively. Grey dashed line and right-hand *y*-axis indicate the ratio of IETD cleavage activity in virus-infected cell extracts relative to control cell extracts.

We observed a dramatic increase in IETD-AMC cleavage only in virus-infected cultures (Fig. 4, black symbols) for both phytoplankton. The ratio of IETDase catalytic activity in CeV-infected *H. ericina* cultures increased from 3-fold at 48 h post-virus addition to 20-fold by 72 h post-virus addition (Fig. 4A, grey dashed line) compared with control cultures. Relative IETDase specific catalytic activity increased from a 1.5-fold difference between *P. pouchetii*, control and PpV-infected cells at 36 h post-virus addition to a 4-fold difference at 72 h post-virus addition (Fig. 4B, grey dashed line).

Pre-treatment of *H. ericina* and *P. poucheti*i cell extracts with z-VAD-fmk for 1 h prior to addition of IETD-AMC confirmed both the biochemical activity and its specificity in these phytoplankton species. The presence of the z-VAD-fmk inhibitor resulted in a 60–80% reduction in IETDase catalytic activity in both control and virus-infected cell extracts (two-tailed *t*-test, all *P* < 0.05, Supplementary data,Fig. S2).

### Effect of *in situ* z-VAD-fmk on virus production

**Fig. 5.**
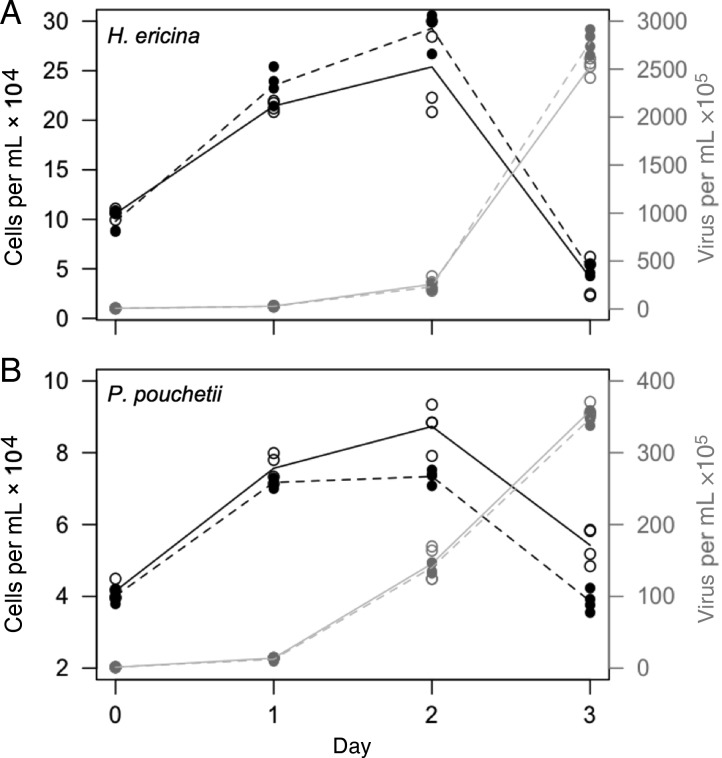
Effect of z-VAD-fmk treatment on growth (black symbols and lines) and virus production (grey symbols and lines) in cultures treated with either 5% (v/v) DMSO (solid circles) or with 20 µM z-VAD-fmk (open circles) cultures of *Haptolina ericina* (**A**) and *Phaeocystis pouchetii* (**B**). Cell numbers (×10 000) are shown on left-hand *y*-axis, while virus numbers (×100 000) are shown on right-hand *y*-axis. *X*-axis indicates days after treatment/infection.

*In situ* addition of z-VAD-fmk to virus-infected cultures of *H. ericina* did not significantly impact host cell growth or CeV production relative to the DMSO control (Fig. 5A, Table I). A small but significant effect of z-VAD-fmk was observed on host cell growth for virus-infected cultures of *P. pouchetii*, as indicated by a slight delay in culture lysis relative to the DMSO control (Fig. 5B, Table I). This growth effect of z-VAD-fmk did not, however, result in a significant effect on virus production (Fig. 5B, Table I) or burst size (DMSO, 628 PpV-01B per cell; z-VAD-fmk, 642 PpV-01B per cell).

**Table I:** Results of general linearized mixed model with penalized quasi-likelihood on culture growth and viral production for (A) Haptolina ericina/CeV-01B and (B) Phaeocystis pouchetii/PpV-01B

Term	Estimate	DF	*t*	*P*
*H. ericina*				
** **Culture growth				
DMSO control	11.487	24	101.56	0.0000
z-VAD-fmk	0.086	12	0.50	0.6234
z-VAD-fmk: Day 1 interaction	−0.171	12	−0.76	0.4640
z-VAD-fmk: Day 2 interaction	−0.233	12	−1.03	0.3217
z-VAD-fmk: Day 3 interaction	−0.357	12	−1.58	0.1403
Virus production (CeV-01B)				
DMSO control	13.402	24	180.56	0.0000
z-VAD-fmk	0.030	12	0.29	0.7787
z-VAD-fmk: Day 1 interaction	0.026	12	0.18	0.8622
z-VAD-fmk: Day 2 interaction	0.061	12	0.41	0.6897
z-VAD-fmk: Day 3 interaction	−0.126	12	−0.85	0.4111
*P. pouchetii*				
Culture growth				
DMSO control	10.599	24	387.42	0.0000
z-VAD-fmk	0.033	12	0.86	0.4072
z-VAD-fmk: Day 1 interaction	0.019	12	0.35	0.7357
z-VAD-fmk: Day 2 interaction	0.140	12	2.55	**0.0253**
z-VAD-fmk: Day 3 interaction	0.304	12	5.56	**0.0001**
Virus production (PpV-01B)				
DMSO control	11.756	24	227.72	0.0000
z-VAD-fmk	0.056	12	0.77	0.4550
z-VAD-fmk: Day 1 interaction	0.083	12	0.81	0.4348
z-VAD-fmk: Day 2 interaction	−0.028	12	−0.28	0.7873
z-VAD-fmk: Day 3 interaction	−0.026	12	−0.25	0.8047

The model was used to test whether z-VAD-fmk treatment of cultures caused significant differences in culture growth or virus production relative to the DMSO negative controls. In addition, the model also compared the DMSO controls with z-VAD-fmk treatments on each experimental day to determine whether there was an interaction between z-VAD-fmk treatment effects and time effects (z-VAD-fmk: day X interaction). Significant differences at the 95% confidence level are underlined.

### Polar lipid analysis

Analytical comparisons of polar lipids in control and CeV-infected cultures of *H. ericina* revealed a relative decrease in glycolipids simultaneous with an increase in phosphatidylethanolamine (PE) lipids in CeV-01B-infected cultures only (Table II). We did not observe any notable differences in the polar lipid profiles of control and PpV-01B-infected *P. pouchetii* cultures, with the exception of a slightly higher concentration of host glycosphingolipids (hGSLs) in control cultures relative to virus-infected cultures (Table II). This class of glycosphingolipids (GSLs) recently reported for *E. huxleyi*, so-called “host” GSLs (hGSLs; Vardi *et al*., 2012), were observed in *P. pouchetii* but not in *H. ericina*. However, no traces of the vGSLs reported for EhV (vGSLs; Vardi *et al*., 2009) were detected in either the control or infected *P. pouchetii* or *H. ericina* cultures. The sialic-acid GSL (sGSL) found in *E. huxleyi* strains (Fulton *et al*., 2014) was not detected either.

**Table II:** Qualitative analysis of intact polar lipids in *Haptolina ericina* and *Phaeocystis pouchetii* cultures during infection by CeV-01B or PpV-01B, respectively

*H. ericina*		Control				CeV infected			
	Days after infection	0	1	2	3	0	1	2	3
Glycolipids^a^	MGDG	+++	+++	+++	+++	+++	+++	nd	nd
	SQDG	++	+++	+++	+++	++	++	nd	nd
	DGDG	+	+	+	++	+	+	nd	nd
Phospholipids	PG	++	++	++	++	nd	++	+++	++
	PE	nd	nd	nd	nd	nd	nd	++	+++
	PC	+++	+++	+++	+++	+++	+++	+++	+++
Betaine lipids	DGTS	++	+	++	++	+	+	nd	nd
	DGTA	nd	nd	nd	nd	nd	nd	nd	nd
	DGCC	nd	nd	nd	nd	nd	nd	nd	nd
Cerebrosides									
Host	hGSL	nd	nd	nd	nd	nd	nd	nd	nd
Viral	vGSL	nd	nd	nd	nd	nd	nd	nd	nd
Sialic acid	sGSL	nd	nd	nd	nd	nd	nd	nd	nd
*P. pouchetii*		Control			PpV infected				
	Days after infection	1	2	3	1	2	3		
Glycolipids	MGDG	+++	+++	+++	+++	+++	++		
	SQDG	+	+	+	+	+	nd		
	DGDG	+	++	++	++	++	+		
Phospholipids	PG	++	++	++	++	++	++		
	PE	++	++	++	++	++	++		
	PC	+	+	+	+	+	tr		
Betaine lipids	DGTS	+	+	+	+	+	+		
	DGTA	nd	nd	nd	nd	nd	nd		
	DGCC	tr	tr	tr	tr	tr	nd		
Cerebrosides									
Host	hGSL	++	+	++	+	+	tr		
Viral	vGSL	nd	nd	nd	nd	nd	nd		
Sialic acid	sGSL	nd	nd	nd	nd	nd	nd		

MGDG, monogalactosyldiacylglycerol; SQDG, sulfoquinosyldiacylglycerol; DGDG, digalactosyldiacylglycerol; PG, phosphatidylglycerol; PE, phosphatidylethanolamine; PC, phosphatidylcholine; DGTS, diacylgycerol-*N*-trimethylhomoserine; diacylglycerylhydroxymethyltrimethyl-fl-alanine; DGCC, diacylglycerylcarboxy-*N*-hydroxymethyl-choline; hGSL, host glycosphingolipid; vGSL, viral glycosphingolipid; sGSL sialic acid sphingolipid.

^a^+++, dominant peak; ++, significant peak, +, minor peak; tr, trace; nd, not detected.

## DISCUSSION

Our investigation of host–virus interactions in the *H. ericina*-CeV and *P. pouchetii*-PpV systems focused on the multi-faceted nature of PCD, given its documented role in regulating the host–virus “arms race” in another haptophyte system (Bidle and Vardi, 2011). Using classical PCD markers, including DNA fragmentation and induction of caspase-like proteolytic activity (Ameisen, 2002), we demonstrate that viral infection induces a PCD-like pathway in *H. ericina* and *P. pouchetii.* Taken together with recent observations in the *E. huxleyi*-EhV system (Bidle *et al*., 2007, 2012; Vardi *et al*., 2009; Bidle and Vardi, 2011), our results suggest this cellular response is conserved in other lineages of marine haptophytes. As such, our findings provide expanded mechanistic insight into the subcellular mechanisms that drive viral-induced mortality of haptophyte hosts by the dsDNA-containing Phycodnaviridae. They are consistent with previous findings of host PCD response to viral infection in the unicellular raphidophyte *Heterosigma akashiwo* (Lawrence *et al*., 2001) and the unicellular chlorophyte *Chlorell*a sp. NC64A (Rose *et al.*, 2009). Our results add to the extensive evidence of PCD occurrence and activation in diverse phytoplankton and its role in the regulation of phytoplankton interactions with biotic and abiotic stressors in the environment.

DNA fragmentation during PCD in higher eukaryotic cells is mediated by endonuclease activity that results in inter-nucleosomal cleavage of chromatin (Peitsch *et al*., 1993; Bortner *et al*., 1995; Nagata, 2000). Here, DNA fragmentation in *H. ericina* and *P. pouchetii* cells was rapid upon virus infection, occurring within 1 day post-virus addition and was also observed in these haptophytes when cells were treated with camptothecin. As such, this chemical treatment served as a useful positive control. The differences in the fragmentation pattern between virus-infected cultures and those treated with camptothecin are notable and likely implicate the potential complexity of host cell machinery that is activated in response to various forms of stress. The weak DNA laddering observed on Days 2 and 3 in control *H. ericina* cultures is likely due to stationary phase cell death in some cells of these cultures, providing further evidence that PCD may be a general stress response in these marine hapotphytes. Nevertheless, the very early (<1 d) stress-phase induction of DNA laddering upon both virus infection and camptothecin treatment suggests that DNA fragmentation may be activated earlier in these haptophytes than in classical metazoan models, in which caspase-initiated DNA fragmentation occurs during later stages of apoptosis (Ameisen, 2002). Similarly, early induction of DNA fragmentation was observed in the cyanobacterium *Plectonema boryanum* (Oscillatoriales) after infection by the LPP-1 virus (Sherman and Haselkorn, 1970). Given our comparatively limited knowledge of the cell biology of these microalgal hosts, in combination with the extensive evolutionary distance to higher metazoans (Berman-Frank *et al*., 2004; Falkowski *et al*., 2004), it is unlikely that the dynamics and expression of stress responses in these unicellular phytoplankton adhere *a priori* to classical timing and cascade pathways shown for higher metazoans.

The difference in DNA fragmentation patterns between virus-infected cultures of *H. ericina* and *P. pouchetii* is noteworthy, and suggests that different nuclease activities or enzymes may be activated by virus infection in these organisms. A search for differential nuclease content in the predicted proteomes of these viruses, however, could not explain this difference (data not shown). The relatively weak intensity of the DNA laddering patterns on agarose gels further suggests that DNA fragmentation only occurs in some host cells, or that only a fraction of host cell chromatin is accessible for endonucleases. It is important to note that *H. ericina* and *P. pouchetii* cultures were not synchronous *sensu stricto* with regard to timing of virus infection, and therefore experiments likely consisted of multiple rounds of infection prior to cumulative culture lysis ∼72 h after virus addition. The persistence of large-molecular weight DNA in control as well as infected cultures may be indicative of un-infected algal cells with intact genomes, infected algal cells with partially fragmented genomes, nascent viral genomes and/or bacterial DNA.

It has been demonstrated for the PBCV-1 virus that type II restriction enzymes are packaged in viral particles and commence hydrolysis of the unmethylated host *Chlorella* sp. genome upon virus infection (Agarkova *et al*., 2006). We identified some potential DNA methylation enzymes in the predicted proteomes of both viruses (Tables III and IV), including one predicted ORF with high amino acid identity to DNA N6-adenine methyltransferases found in other large viruses. For both CeV-01B and PpV-01B, we were only able to identify one endonuclease-like protein-coding ORF with highest amino acid similarity to a repair nuclease gene found in the *P. globosa* virus PgV genome sequence (Table IV, accession number YP_008052636.1). We are not aware of any such enzymes or accessory proteins that are embedded in CeV-01B or PpV-01B virus particles; to date the proteomes of mature CeV-01B and PpV-01B virions have not been determined.

**Table III:** Results of blastp analysis of predicted protein-coding ORFs in the CeV-01B and PpV-01B draft genome sequences that were found to contain canonical tetrapeptide caspase recognition sequences

Caspase	Motif	Blastp hit	Accession number	*e*-value	Identities
CeV-01B					
4	LEVD	Hypothetical protein [Bacteria]	WP_018003408.1	2e−25	113/374
4	LEVD	dTDP-glucose 4,6-dehydratase [*Strigomonas culicis*]	EPY35950.1	1e−99	158/320
PpV-01B					
4	LEVD	Hypothetical protein PGAG_00002 [*Phaeocystis globosa* virus 12T]	AET72892.1	1e−70	213/514
4	LEVD	Hypothetical protein PGAG_00002 [*Phaeocystis globosa* virus 14T]	AET73710.1	7e−26	114/362
4	LEVD	Hypothetical protein PGAG_00002 [*Phaeocystis globosa* virus 12T]	AET72892.1	0	501/1036
4	LEVD	Hypothetical protein PGCG_00002 [*Phaeocystis globosa* virus]	YP_008052361.1	8e−53	145/344
4	LEVD	Hypothetical protein [*Oscillatoria* sp. PCC 10802]	WP_017718387.1	0.002	38/103
4	LEVD	Hypothetical protein PGAG_00002 [*Phaeocystis globosa* virus 12T]	AET72892.1	5e−87	283/766
4	LEVD	Lambda-type exonuclease [*Phaeocystis globosa* virus]	YP_008052447.1	0	407/474
5/9	LEHD	hypothetical protein PGCG_00246 [*Phaeocystis globosa* virus]	YP_008052564	0	283/318
5/9	LEHD	Put. membrane protein EhV146 [*Emiliania huxleyi* virus 86]*	YP_293899.1	2e−25	53/62
6	VEID	Hypothetical protein [*Emiliania huxleyi* virus 99B1]*	CAZ69539.1	2e−88	133/137
7	DEVD	Hypothetical protein BpV2_130 [*Bathycoccus* sp. RCC1105 BpV2]	ADQ91297.1	6e−75	136/316
7	DEVD	Heat shock protein 70 [*Cladospoium herbarum*]	AAB47209.1	9e−41	74/159
7	DEVD	Hypothetical protein PGCG_00262 [*Phaeocystis globosa* virus]	YP_008052580.1	0	836/1048
8	IETD	Polynucleotide kinase-3′-phosphatase [*Phaeocystis globosa* virus]	YP_008052398.1	4e−176	241/285
8	IETD	DNA-directed RNA pol II subunit RPB5 [*Phaeocystis globosa* virus]	YP_008052525.1	6e−88	129/151

For each ORF are given the representative caspase-family enzyme number, the canonical tetrapeptide cleavage motif for that caspase, a description of the best blastp hit against the NCBI non-redundant protein database, and the accession number, *e*-value and number of amino acid identities for the best blastp hit. Asterisks indicate highest similarity to predicted *Emiliania huxleyi* virus proteins containing caspase cleavage sites (Bidle *et al*., 2007).

**Table IV:** Predicted proteins in the draft genomes of CeV-01B and PpV-01B with blastp amino acid similarity to endonucleases or restriction-modification enzymes

Blastp hit	Accession number	*e*-value	Identities
CeV-01B			
Type I restriction-modification protein subunit M [*Microscilla marina*]	WP_002701743.1	3e−11	48/142
Adenine-specific DNA methyltransferase [*Phaeocystis globosa* virus]	YP_008052748.1	2e−69	104/33
DNA methyltransferase [*Phaeocystis globosa* virus]	YP_008052723.1	2e−18	45/94
Methyltransferase FkbM [*Limnobacter* sp. MED105]	WP_008250363.1	2e−23	48/101
ERCC4-type DNA repair nuclease [*Phaeocystis globosa* virus]	YP_008052636.1	9e−46	106/258
PpV-01B			
Cytosine-specific methyltransferase [*Ostreococcus tauri* virus 2]	YP_004063436.1	3e−139	204/314
Adenine-specific DNA methyltransferase [*Phaeocystis globosa* virus]	YP_008052748.1	6e−172	235/272
Putative DNA N6-adenine methyltransferase [*Cafeteria roenbergensis* virus BV-PW1]	YP_003969980	3e−51	89/141
Putative DNA N6-adenine methyltransferase [*Cafeteria roenbergensis* virus BV-PW1]	YP_003969980	7e−41	84/143
ERCC4-type DNA repair nuclease [*Phaeocystis globosa* virus]	YP_008052636.1	2e−130	200/253

For each predicted protein are given a description of the best blastp hit to the NCBI non-redundant protein database, and the accession number, *e*-value and number of amino acid identities for each blastp hit.

The *E. huxleyi* genome sequence contains nine metacaspase genes (Read *et al*., 2013), ancestral orthologues of caspases (Uren *et al*., 2000; Carmona-Gutierrez *et al*., 2010). The existence of caspase-family proteins in *H. ericina* and *P. pouchetii* is unknown. Although the proteins responsible for the observed caspase activities in *H. ericina* and *P. pouchetii,* and indeed in all other phytoplankton, still remain largely unknown, biochemical tools exist to determine whether analogous enzymatic catalytic activity is present in cells and cell extracts (Vardi *et al*., 1999, 2009; Berman-Frank *et al*., 2004; Evans *et al*., 2006; Bidle *et al*., 2007, 2010; Bidle and Bender, 2008; Thamatrakoln *et al*., 2011). The canonical tetrapeptide substrate IETD-AMC, in *H. ericina* and *P. pouchetii* cell extracts provided additional biochemical evidence for virus-induced PCD activation. This activity could be specifically inhibited by treatment with the pan-caspase inhibitor, z-VAD-fmk, thereby verifying the biochemical specificity of this activity, but did not reduce viral production during infection of either *H. ericina* or *P. pouchetii.* This differentiates it from the *E. huxleyi*-EhV system (Bidle *et al*., 2007), in which such *in vivo* caspase inhibition largely abolished (∼90%) EhV production, indicating a dependence on this activity for productive infection and invoking a Red Queen host-virus co-evolutionary dynamic around this activity and PCD (Van Valen, 1973; Bidle and Vardi, 2011). A conserved utilization of host caspase-like activity by viruses for successful replication would argue for the evolution of viral replication strategies to “hijack” this host-derived enzymatic activity (Bidle *et al*., 2007) and/or to modulate host PCD (Bruchhaus *et al*., 2007; Vardi *et al*., 2009) for their own benefit. Here, we demonstrate that virus infection of *H. ericina* and *P. pouchetii* induces host caspase-like activity and a PCD-like phenotype that is independent of a need for viral propagation. These findings suggest that the observed increase in caspase-like activity is a characteristic response of *H. ericina* or *P. pouchetii* to infection*,* but it is not essential for normal viral infection dynamics. It should be noted that the algal cultures used in this study were not axenic, and therefore we cannot conclusively rule out that bacterial activity influenced the results.

One of the primary control points in the *E. huxleyi*-EhV haptophyte system rests in the viral regulation of lipid production, especially a diverse suite of glycosphingolipids (Vardi *et al*., 2009, 2012; Bidle and Vardi, 2011; Fulton *et al*., 2014; Rose *et al*., 2014), which are powerful inducers of PCD in eukaryotes (Hannun and Obeid, 1995). We investigated whether similar lipid-based dynamics were at play in these systems, but consistent with the results of Maat *et al*. (Maat *et al*., 2013), we were unable to identify differences in polar lipid profiles between control- and virus-infected cultures.

Serine palmitoyl transferase (SPT) is the enzyme that catalyses the first and rate-limiting step in the biosynthesis of GSLs (Hanada, 2003). The draft genome sequence of PpV-01B also contains two putative serine palmitoyltransferase gene precursors (H. Ogata, unpublished data). However, the fact that none of the other genes required for sphingolipid biosynthesis are present in the draft PpV-01B genome corroborates our inability to detect vGSL production in *P. pouchetii* during PpV infection, despite our detection of host GSLs, and suggests that these polar lipids are likely not involved in the *P. pouchetii*–PpV interaction.

During CeV infection of *H. ericina*, we did identify a notable increase in PE lipids in CeV-infected cultures. PE lipids comprise one of the major structural lipid groups in eukaryotic membranes (Van Meer *et al*., 2008; Martin *et al*., 2011; Maat *et al*., 2013), although they can also be found in marine prokaryotes (Popendorf *et al*., 2011). It is unclear whether the increase in PE lipids in the CeV-infected *H. ericina* culture is a consequence of production of host lipids for viral packaging (Maat *et al*., 2013) or a consequence of bacterial proliferation due to release of dissolved organic matter after viral lysis. PE-containing heterotrophic marine bacteria almost invariably also contain phosphatidylglycerol (Oliver and Colwell, 1973; Van Mooy *et al*., 2009; Popendorf *et al*., 2011), and as increases in phosphatidylglycerol were not observed we believe that the increase in PE is associated with viral infection. This idea is further supported by observations of induced PE synthesis in EhV86-infected *E. huxleyi* cells, which could not be specifically attributed to bacteria (Fulton *et al*., 2014). The decrease in glycolipids, which are likely located in the chloroplast, in CeV-01B-infected cultures of *H. ericina*, parallels that of the decrease in Fv/Fm. This decrease may likely have been due to disruption of infected cells during the filtration process, allowing passage of organelles, including chloroplasts, through the GFF filters and thereby precluding their detection in polar lipid analyses (J. L. Ray, personal observation). The authors are unaware of any studies of algal host–virus systems in which the photophysiological effects of viral infection have been shown to be due to a fundamental disruption of the thylakoid membranes in algal host cells.

Examination of the EhV86 genome provided diagnostic evidence that successful viral infection requires host caspase activity for viral protein processing (Bidle *et al*., 2007). In order to assess potential co-evolutionary dependencies in our host–virus systems, we analysed the draft proteomes of CeV-01B and PpV-01B (H. Ogata, Marseille, unpublished data) for proteins that contained internal, classic caspase tetrapeptide recognition sequences (Thornberry, 1998) (Supplementary data). Analysis of the draft CeV-01B proteome indicated only two ORFs containing a putative caspase cleavage site (LEVD), whereas the draft proteome of PpV-01B contains 15 predicted protein-coding genes, most of which had highest sequence similarity to hypothetical viral proteins in the NCBI database with known tetrapeptide caspase cleavage motifs. Two of the predicted PpV-01B proteins containing caspase cleavage motifs had highest similarity to predicted proteins in the EhV genome with identical caspase cleavage motifs (Bidle *et al*., 2007). Clearly, the predicted proteome of the PpV-01B virus suggests the potential for co-evolutionary dependence upon host caspase activity for protein processing during viral replication, as is the case for the *E. huxleyi*-EhV system, although the specific activity that may mediate this host–virus interaction cannot be inhibited by z-VAD-fmk treatment.

The marine haptophytes investigated here, which are both infected by members of the Phycodnaviridae, differ in key ecological properties. *Haptolina ericina* is not known to form seasonal blooms despite its ubiquity (Sandaa *et al*., 2001), while the annual spring bloom of *P. pouchetii* is a well-documented phenomenon (Schoemann *et al*., 2005). Even the two bloom-forming algae *P. pouchetii* and *E. huxleyi* display fundamental differences with the respect to life-cycle properties. Spring *P. pouchetii* blooms consist of gelatinous colonies containing many hundreds to thousands of cells, while blooms of *E. huxleyi* consist strictly of single cells. The activation of a PCD-like pathway during virus infection in these ecologically diverse phytoplankton implicates the conservation of genetic cellular programmes among unicellular eukaryotes in response to stress in the marine environment. Although the PCD-like response of the haptophytes *E. huxleyi*, *H. ericina* and *P. pouchetii* to virus infection is common among them, our results highlight differences between the *H. ericina*-CeV and *P. pouchetii-*PpV systems investigated in this study, from the *E. huxleyi*-EhV model of host–virus interaction (Bidle and Vardi, 2011).

The accumulating body of evidence documenting phytoplankton PCD responses to both biotic and abiotic stress have begun to unveil its potential to influence how primary producers interact with their environment and regulate fitness and diversity. Despite considerable attention to PCD-like pathways in a diversity of marine microeukaryotes (Bidle and Falkowski, 2004; Franklin *et al*., 2006), we still have an insufficient mechanistic understanding of their regulation and their evolution and function in the broader context of complex aquatic microbial ecosystems. In this study, we provide evidence that PCD-like mechanisms exist in diverse marine haptophytes and that these mechanisms are consistently triggered by viral infection. Consequently, these data add an important piece to our growing understanding of stress response mechanisms in marine haptophytes.

## SUPPLEMENTARY DATA

Supplementary data can be found online at http://plankt.oxfordjournals.org.

## FUNDING

Funding to J.L.R, R.-A.S. and A.L. was provided by the Norwegian Research Council for the “VIPMAP” (nr. 186142) and “HAPTODIV” (nr. 190307) projects, and by the European Research Council Advanced Grant ERC-AG-LS8 “Microbial Network Organisation” (MINOS, project number 250254). J.L.R. received a FRIBIO overseas research fellowship from the Norwegian Research Council. K.D.B. and B.V.M. were supported by funding from the United States National Science Foundation (OCE-1061883). Funding to pay the Open Access publication charges for this article was provided by the Bergen Open Research Archive (BORA) through the University of Bergen Library.

## Supplementary Material

Supplementary Data
