# Supplementary material for: Virus infection of Haptolina ericina and Phaeocystis pouchetii implicates evolutionary conservation of programmed cell death induction in marine haptophyte–virus interactions
Source: J Plankton Res. 2014 May 5;36(4):943–55. doi: 10.1093/plankt/fbu029 (PMC4090681; doi:10.1093/plankt/fbu029)
Supplement: Supplementary Data [file supp_36_4_943__index.html]

Virus infection of Haptolina ericina and Phaeocystis pouchetii implicates evolutionary conservation of programmed cell death induction in marine haptophyte–virus interactions — Virus infection of Haptolina ericina and Phaeocystis pouchetii implicates evolutionary conservation of programmed cell death induction in marine haptophyte–virus interactions — Supplementary Data 

# Virus infection of *Haptolina ericina* and *Phaeocystis pouchetii* implicates evolutionary conservation of programmed cell death induction in marine haptophyte–virus interactions

## Supplementary Data

Supplementary Data

**Files in this Data Supplement:**

- Supplementary Data - Doc file
- Supplementary Figure 1 - jpg file
- Supplementary Figure 2 - jpg file
