# Supplementary material for: Virus infection of Haptolina ericina and Phaeocystis pouchetii implicates evolutionary conservation of programmed cell death induction in marine haptophyte–virus interactions
Source: J Plankton Res. 2014 May 5;36(4):943–55. doi: 10.1093/plankt/fbu029 (PMC4090681; doi:10.1093/plankt/fbu029)
Supplement: Supplementary Data [file supp_fbu029_fbu029supp.doc]

**Supplementary Information**

Virus infection of *Haptolina ericina* and *Phaeocystis pouchetii* implicates evolutionary conservation of programmed cell death induction in marine haptophyte-virus interactions

Jessica L. Ray^1,2^*, Liti Haramaty^3^, Runar Thyrhaug^2✝^, Helen Fredricks^4^, Benjamin A. S. Van Mooy^4^, Aud Larsen^1,2^, Kay D. Bidle^3^, Ruth-Anne Sandaa^2^

^1^Uni Environment, Uni Research AS, Thormøhlensgt 49B, N-5006 Bergen, Norway

^2^Department of Biology, University of Bergen, Thormøhlensgt 53A, N-5006 Bergen, Norway

^3^Institute of Marine and Coastal Sciences, Rutgers University, 71 Dudley Road, New Brunswick, NJ 08901, USA

^4^Marine Chemistry and Geochemistry, Woods Hole Oceanographic Institution, Woods Hole, MA 02543 USA

^✝^ deceased

**Methods**

*IETDase catalytic activity*

Frozen cell pellets were thawed briefly on ice and 100-200 µl of sonication buffer (50 mM Hepes, pH 7.3, 100 mM NaCl, 10% w/v sucrose, 0.1% w/v CHAPS, 10 mM dithiothreitol) (Lauber et al. 2001) was added to each pellet. Samples were sonciated on ice twice for 20 sec with rest on ice in between sonciations to keep samples cold. Sonicates were immediately centrifuged at 18000 *x g* at 4°C for 5 min to pellet insoluble proteins and cell debris. Supernatants containing soluble proteins were transfered to new tubes and utilised for further analysis. Cell extracts were assayed in duplicate for protein content using a biocinchoninic acid (BCA) assay (Pierce/Thermo Scientific, Rockford, IL), according to the manufacturer's instructions. Colorimetric quantitation of protein in the BSA standards and samples was performed using a Molecular Devices multi-mode plate reader. An equal volume from all cell extract samples was assayed for isoleucyl-glutamyl-threonyl-aspartic acid-7-amino-4-methylcoumarin (IETD-AMC, Calbiochem, Darmstadt, Germany) catalytic cleavage activity in triplicate in black flat-bottom microtiter plates. For each sample, a master mix of sample (usually 20-100 µl), 10 mM dithiothreitol and sonication buffer (q.s. 420 µl) was prepared in a sterile microcentrifuge tube. One hundred microlitres was then pipetted into each of four wells in the assay plate. Three of the four sample wells received 20 µM IETD-AMC. The fourth well received no substrate and was included as a sample blank to measure background sample fluorescence in the absence of fluorogenic substrate addition. Three wells in the assay plate received sonication buffer and substrate but no sample, and served as substrate blanks to measure background fluorescence of the substrate and buffer in the absence of biological substrate cleavage activity. The plate was incubated at 26°C in a SpectraMax Gemini XS plate reader (Molecular Devices, Sunnyvale, CA) and fluorescence (excitation 400 nm, emission 505 nm) was measured for each well every 10-20 min for up to several hours. Caspase activity was determined from the highest fluorescence read time point at which fluorescence increase over time was still linear for all wells, then normalized to relative fluorescence units (RFU) mg protein-1 hr-1 (Bidle et al. 2007).

*Burst size*

Measurement of variation in burst size is a means to quantify the effects of treatments on viral fitness (Hellweger 2009). For normal infection of *C. ericina* by CeV, we calculated a burst size between 700-1400, which fits within the reported range for this host-virus system (Sandaa et al. 2001). For *P. pouchetii*, we observed burst sizes between 140 and 720 PpV for different experiments. These values are within the range of burst sizes previously reported for PpV infection of *P. pouchetii* (Jacobsen et al., 1996; Thyrhaug et al., 2002).

*Camptothecin treatment*

Camptothecin is a known inhibitor of DNA topoisomerase I, inducing apoptotic PCD in eukaryotic cells through accumulation of double-strand DNA breaks and subsequent destabilisation of mitochrondrial membranes due to oxidative stress (Sen et al., 2004). The ability of camptothecin to induce PCD markers in *H. ericina* and *P. pouchetii* was tested by treating two-liter cultures with 2 µM camptothecin, and sampling for DNA fragmentation analysis.

*In silico identification of potential caspase cleavage sites in draft virus genomes*

Predicted protein-coding open reading frames (ORFs) present in the draft genome sequences of CeV-01B and PpV-01B (H. Ogata, unpublished results) were searched for various canonical caspase tetrapeptide cleavage sites: YVAD (caspase-1), VDVAD (caspase-2), DEVD (caspase-3), LEVD (caspase-4), WEHD (caspase-5), VEID (caspase-6), IETD (caspase-8 and others), and LEHD (caspase-9) using a Perl script (S. Modha, Leicester Univ, UK). Candidate ORFs containing a cleavage site were further analysed by *blastp* (Altschul et al., 1997) searches against the NCBI non-redundant protein database.

**References**

Altschul, S. F., Madden, T. L., Schäffer, A. A., Zhang, J., Zhang, Z., Miller, W. and Lipman, D. J. (1997) Gapped BLAST and PSI-BLAST: a new generation of protein database search programs. *Nucl. Acids Res.,* **25**, 3389--3402.

Bidle, K. D., Haramaty, L., Ramos, J. B. E. and Falkowski, P. G. (2007) Viral activation and recruitment of metacaspases in the unicellular coccolithophore, *Emiliania huxleyi*. *Proc. Nat. Acad. Sci. USA,* **104**, 6049--6054.

Hellweger, F. L. (2009) Carrying photosynthesis genes increases ecological fitness of cyanophage in silico. *Environ. Microbiol.,* **11**, 1386--1394.

Jacobsen, A., Bratbak, G. and Heldal, M. (1996) Isolation and characterization of a virus infecting *Phaeocystis pouchetii* (Prymnesiophyceae). *J. Phycol.,* **32**, 923--927.

Lauber, K., Appel, H. A. E., Schlosser, S. F., Gregor, M., Schulze-Osthoff, K. and Wesselborg, S. (2001) The adapter protein apoptotic protease-activating factor-1 (Apaf-1) is proteolytically processed during apoptosis. *J. Biol. Chem.,* **276**, 29772--29781.

Sandaa, R.-A., Heldal, M., Castberg, T., Thyrhaug, R. and Bratbak, G. (2001) Isolation and characterization of two viruses with large genome size infecting *Chrysochromulina ericina* (Prymnesiophyceae) and *Pyramimonas orientalis* (Prasinophyceae). *Virol.,* **290**, 272--280.

Sen, N., Das, B. B., Ganguly, A., Mukherjee, T., Tripathi, G., Bandyopadhyay, S., Rakshit, S., Sen, T. and Majumder, K. (2004) Camptothecin induced mitochondrial dysfunction leading to programmed cell death in unicellular hemoflagellate *Leishmania donovani*. *Cell Death Diff.,* **11,** 924--936.

Thyrhaug, R., Larsen, A., Brussaard, C. P. D. and Bratbak, G. (2002) Cell cycle dependent virus production in marine phytoplankton. *J. Phycol.,* **38**, 338--343.

**Supplementary figure legends**

Figure S1. Induction of DNA fragmentation during treatment of *Haptolina ericina* and *Phaeocystis pouchetii* cultures with 2 µM camptothecin. Lane identifiers: M, dsDNA molecular weight marker with sizes shown in basepairs; A, positive control U937 apoptotic cells; 0-24, time in hours after addition of 20 µM camptothecin to 2L cultures.

Figure S2. *In vitro* inhibition of IETD cleavage activity in soluble cell extracts of control or virus-infected cultures of *H. ericina* (grey bars) or *P. pouchetii* (white bars) using the pan-caspase inhibitor z-Val-Ala-Asp-fluoromethyl-ketone (z-VAD-fmk). Percent reduction, relative reduction in IETD cleavage activity when cell extracts were pre-treated with 20 µM z-VAD-fmk prior to IETD cleavage assay, versus the same cell extracts without z-VAD-fmk pre-treatmnent.
