# Supplementary figures and images for: Virus infection of Haptolina ericina and Phaeocystis pouchetii implicates evolutionary conservation of programmed cell death induction in marine haptophyte–virus interactions
Source: J Plankton Res. 2014 May 5;36(4):943–55. doi: 10.1093/plankt/fbu029 (PMC4090681; doi:10.1093/plankt/fbu029)

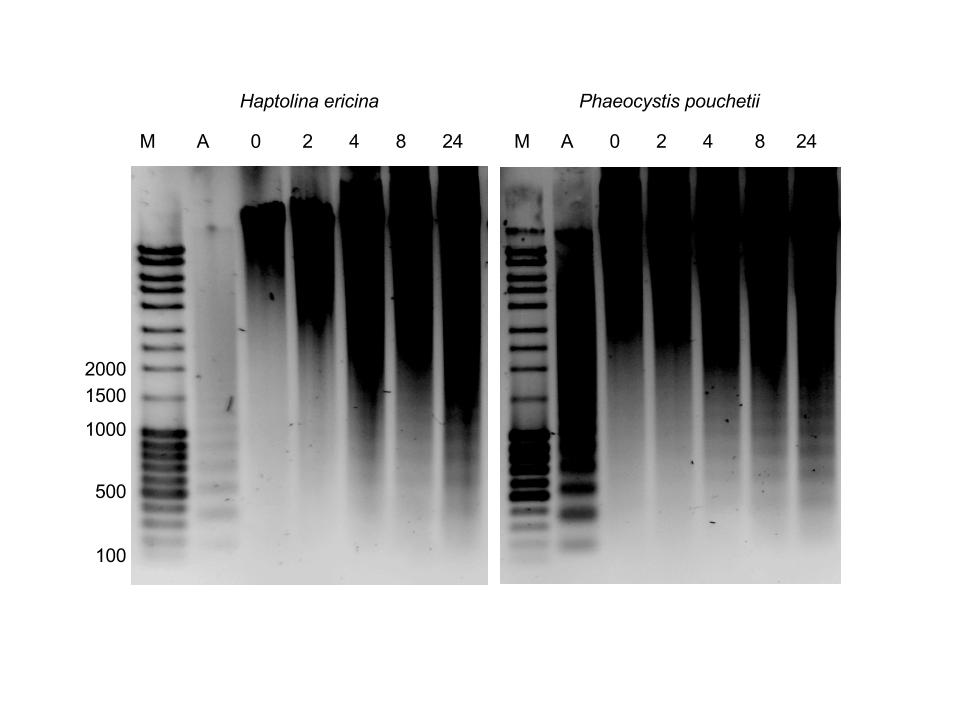

Supplement: Supplementary Data [file supp_fbu029_fbu029supp_fig1.jpg]

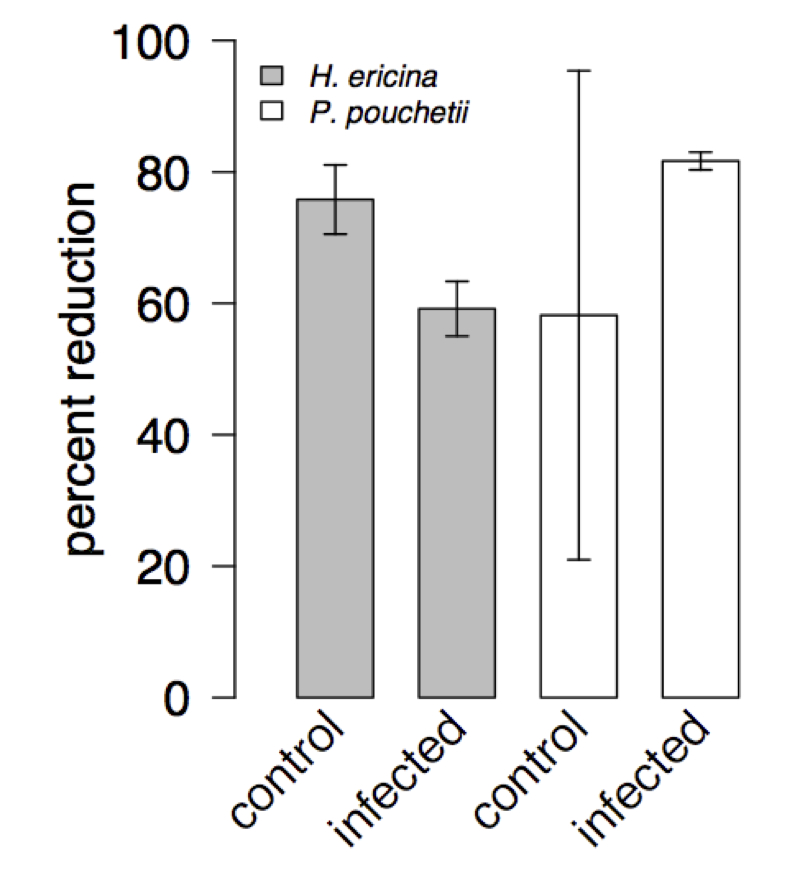

Supplement: Supplementary Data [file supp_fbu029_fbu029supp_fig2.jpg]
